# American Medical Association

**Published:** 1896-06

**Authors:** 


### SOCIETY REPORTS.

### AMERICAN MEDICAL ASSOCIATION.

ATLANTA, GA., May 15, 1896.

The Forty-Seventh Annual Meeting of the Association was held in Atlanta, May 5th, 6th, 7th, and 8th. The Association assembled in DeGive's Opera House, May 5th, at 10 o'clock, and was called to order by the President, Dr. R. Beverly Cole, of California. After prayer by Dr. Henry McDonald, the Addresses of Welcome were delivered by Dr. Frank M. Ridley of LaGrange, and Hon. John Temple Graves of Atlanta.

Announcements were then made by the chairman of the Committee of Arrangements, Dr. Willis F. Westmoreland, after which the president delivered his address.

Dr. Cole stated that the American Medical Association had, within the past two years, brought about great changes in the desired direction of higher education. It must, however, be admitted that improvement had been effected but very slowly, and that the usage of many of the schools to evade the laws established by the Association is so general, that the good result is small, and the manifest reluctance of so many now within the organization to embrace the last advance, namely, the adoption of the four years' requirement, gives but little promise for the future. Just so long as the examinations for matriculation are conducted by members of the faculty of medical schools, just so long will evils continue; and so long as the professional examinations for degrees are conducted by interested parties, just so long will the ranks of the noblest profession be filled with uneducated, untrained, so-called doctors.

Another question of grave importance, and to which he thought the Association should take cognizance and suggest a remedy, was the total absence of reciprocity between the United States and foreign countries as to laws governing the right to practice. Why Americans should be required, when taking up their abode in Germany, Great Britain, and even in the territory of our first cousin, Canada, to undergo an examination preliminary to securing a license, while our portals are floodgates through which every country of the earth pours its surplus of medical men; or rather, to put it more correctly, why our country should receive with open arms, without hindrance, the excess of product of foreign schools without requiring of them the same as is required of us, he could not see, and he was distinctly of the opinion that the time had arrived when the matter should be earnestly considered and something done to arrest the strides of this rapidly growing wrong.

Equally gratifying is the effort now being made asking Congress for an additional member of the Cabinet, who shall be known as the Secretary of Public Health, and who shall be head of a department to be known as the Bureau of Health, which will have general charge of health matters as well as statistics. Such a department would be of incalculable utility and value, and the measure should by all means possible be vigorously pushed forward.

The Address on Medicine was delivered by Dr. William Osler, of Baltimore, upon

THE STUDY OF THE FEVERS OF THE SOUTH.

Humanity has but three great enemies: fever, famine, and war, of which by far the greatest, by far the most terrible, is fever. It is worthy of comment that three of the greatest benefits conferred on mankind, beside which it would be hard to name three of equal importance, have been in connection with the fevers, the introduction of cinchona, the discovery of vaccination, and the announcement of the principle of asepsis. The differentiation of special forms of the continued fevers, and particularly that of typhoid, forms one of the most interesting chapters in medicine.

It is a very gratifying sign to notice the attention which has been given of late to the subject of typhoid fever in the South. Some years ago a good many physicians resented the imputation that the disease, to any extent, prevailed in the Gulf States.

He had been in the habit for several years of reading the reports of the discussions on this subject at the New Orleans Parish Medical Society, and they have been interesting as showing a progressive development of knowledge such as comes to all of us with fuller study of any problem. Enteric fever presents no constant picture. On the contrary, scarcely any disease has a more varied symptomatology. The fever may be said to be invariable, though afebrile cases are not unknown; but in the features of onset, in the length of its course, in the presence or absence of symptoms regarded as cardinal, such as rose spots, diarrhea and splenic enlargement, typhoid fever is so uncertain that the diagnosis is often dubious.

Advances in the treatment of fevers, and especially of typhoid, have not kept pace with the rapid progress in our knowledge of the etiology. Think of the misery, the tediousness, the discomfort of a typhoid case with three relapses; think of the bleeding, the blistering, the purging, from which at least our fever patients of to-day are free. Contrast with the former methods the care, the gentle nursing, the scrupulous cleanliness, the abundance of cold water to drink, and fresh air which typhoid fever patients of to-day receive.

Full clinical histories should be *first* furnished of typhoid fever cases, and any one who wishes to contribute to the subject should not be too busy, not only to make a careful, critical study of the symptoms, but to jot them down in some order so that at least they may be intelligible to others. The *second* point is the necessity of obtaining autopsies in fatal cases. We all appreciate how difficult this is in private practice, but in determining the nature of obscure typical cases of fever it is absolutely essential. There is not a hospital in the country in which the determination of the nature of an obscure case of fever is not settled by the autopsy alone. *Thirdly*, it is essential that observers who undertake to study this question with thoroughness should approach it with a full acquaintance with the varieties of the malarial parasites, and with an accurate knowledge of the bacteriological technic.

To us, as a profession, belongs the chief glory of the century. Enormous as has been the advancement in material prosperity, and widespread as has been the diffusion of benefits from the development of the physical sciences, they cannot compare with the progress which has been made in the relief of suffering and in the prevention of disease. Our work here ranks among the most memorable achievements in the history of the race. Fever in its various forms is still with us, and the century has seen in connection with it but one discovery of the first magnitude, but it is almost of equal importance to know that the way has been opened, and that the united efforts of many workers in many lands are day by day disarming this great enemy of the race.

Dr. Nicholas Senn, of Chicago, delivered the Address on Surgery, choosing for his subject

### SOME OF THE LIMITS OF THE ART OF SURGERY.

Modern surgery has attained a degree of development which entitles it to the distinction of a science and an art. As a science, surgery is of recent date, having been founded and perfected during the last half of the present century. As an art it has been practiced for centuries by our ancestors, with credit to themselves and benefit to the injured, the crippled, and the sick. When Boyer wrote the introduction to his classic work on surgery, he expressed the conviction that surgery had reached perfection. How little did he dream of the great changes that would be wrought in the practice of his cherished profession by the progressive pathologists and surgeons of the next few generations.

The *furor operativus* manifested in special departments of surgery, and its obvious results, render the standing and legitimate scope of the general surgeon very uncertain and indefinite at the present time. Let the general surgeon turn to the right or to the left, advance or retreat, and he finds himself on reserved territory. As for the physician, he is expected to answer night calls, prescribe for diarrhea and whooping-cough, watch cases of typhoid fever, measles, scarlatina and smallpox, and should complications arise, and he does not report to the proper authority, he renders himself liable to censure. Much of this ill-applied energy in the surgical world has resulted in detriment to patients and in retarding actual surgical progress. Operative surgery has been carried to extremes.

Operative interference is absolutely indicated in fractures of the cranial vault under the following circumstances: (1) All open fractures, including gunshot and punctured fractures. (2) Depressed fractures, attended by well-defined symptoms, caused either by the depression or intracranial complications. (3) Rupture of the middle meningeal artery, with or without fracture of the skull. The indiscriminate use of the chisel and the trephine in the hands of the inexperienced practitioner is fraught with danger, and should not be encouraged by teachers and expert surgeons. Cerebral localization and aseptic surgery have made it possible to treat a few intracranial lesions successfully by direct operative interference.

The abdominal cavity was largely a *terra incognita* to the surgeon of less than half a century ago. To-day it is the favorite battle-ground of the average surgeon, and the select field of the so-called abdominal surgeon. Notwithstanding the wonderful improvements in the technic of operations upon the stomach, partial gastrectomy and pilorectomy have yielded anything but encouraging results. In nearly fifty per cent. the patients subjected to radical treatment for malignant disease of the stomach succumbed to the immediate effects of the operation. The speaker has opened the abdominal cavity for the surgical treatment of malignant disease of the stomach nineteen times, and only in one case did he find the disease limited to the organ first affected, and in this case the general health of the patient had been so much deteriorated by the obstructive pyloric carcinoma as to contraindicate radical operation; in all of the remaining cases a pylorectomy or partial gastrectomy was out of the question, as the carcinoma of the pylorus or stomach had extended to adjacent organs, or had given rise to regional infection through the lymphatic glands sufficiently to contraindicate any attempts at radical removal of the disease.

The greatest onslaught of modern surgery has been upon the organs of generation, male and female. The future historians who will record the work of many gynecologists belonging to the present generation will have reason to express their surprise at what disasters the art of surgery has produced when plied in cases far in advance of a scientific foundation. Here and there we hear a feeble voice protesting against the indiscriminate surgery upon the organs of generation of the opposite sex, but the mutilating work continues in spite of such opposition and well-meant advice. When we arraign the gynecologists before such a representative body, composed of the representative medical men of the country, for innumerable and inexcusable transgressions of the rules which ought to govern and control the art of surgery, we do not include the scientific, conscientious workers in that department of surgery, but our remarks apply to a class of routine operators which has recently grown to alarming dimensions, not only in this but in nearly every country penetrated by the dim rays of so-called bold surgery. The new generation of doctors finds no longer satisfaction in practicing their profession in some rural district. They have their eyes on large cities and have heard of enticing fees paid to specialists for insignificant operations. The recent graduate, or the man who has become disgusted with country practice, sees a much-employed gynecologist, follows his work for a month or two, and returns to his prospective field of labor a full-fledged specialist.

The frequency with which women are being castrated is one of the most flagrant transgressions of the limits of the art of surgery. It is not unusual for one operator to exhibit five or six normal ovaries as the result of half a day's work. All kinds of excuses are made for this kind of surgery. Where is this wholesale unsexing of our female population going to end? The beginning of the end has come. The army of women minus their essential organs of generation is beginning to raise its voice against such mutilating work. The number of women who willingly sacrificed their ovaries to restore their shattered health without securing the expected relief has increased to an alarming extent. This sad experience has made the gynecologists more desperate and bold. It is difficult to say where this rage for the removal of the female sexual organs will end, or what organ will be the next battle-ground for the aggressive gynecologists. The clitoris, the vagina, the cervix uteri, the ovaries, the Fallopian tubes, the uterus and its ligaments, have successively passed through a trying ordeal of operative furor. What the next fad will be it is impossible to foretell.

The address on State Medicine was delivered by George H. Rohe, M.D., of Baltimore, subject,

### THE PURIFICATION OF WATER SUPPLIES.

The most vitally important sanitary problem confronting American municipalities at the present day is, unquestionably, the supply of pure water for drinking and other domestic purposes. The widespread prevalence of typhoid fever may be practically looked upon as a result of the pollution of the drinking water. The importance, therefore, of having the latter of a pure quality is self-evident. In 1894 twenty-five of the principal cities of the United States had an average typhoid mortality of 39.6 per one hundred thousand of their population.

Pure water, or water free from all sorts of uncleanness, is demanded to-day by the "sanitary conscience" of the public. We are therefore reduced to one of two alternatives: either to prevent the access of impurities to the sources of supply, or else to resort to some method of purification of the water itself. The city of New York has recently chosen the first alternative by purchasing large tracts of land bordering upon its source of water supply. By the removal of sources of pollution from the area of land so acquired the endeavor has been made to secure a pure drinking water. But the expense has been something enormous.

In sand filtration two processes must be kept in view: one, the straining out of the bacteria, and the other the conversion of the organic matter into inorganic compounds. These imply that the materials of which the filter is composed shall be sufficiently fine to hold back all suspended matter, and that a sufficient supply of oxygen shall always be present to oxidize it. In most natural waters there is a sufficiently large quantity of free oxygen present to cause the oxidizing processes to go on continually, but in extreme cases of pollution an extra supply of oxygen is necessary. This may be supplied by aëration of the water before filtration, or by conducting the filtration intermittently.

### THE JENNER CENTENARY.

The character of Dr. Jenner and the history of his discovery of the protective value of vaccination were the subjects of the first address. In the absence of its author, Dr. N. S. Davis, the paper was read by Dr. Didama, of New York State. The paper recounted the well known facts concerning the life of Jenner and the manner of his great discovery, the story being told in an interesting way.

Dr. H. R. Storer, of Rhode Island, gave a list of the Jenner memorial medals, tablets, portraits, etc.

Dr. Geo. M. Sternberg, Surgeon-General United States Army, read a paper entitled, "Scientific Researches Relating to the Specific Infectious Agent of Smallpox and the Production of Artificial Immunity from this Disease."

Dr. Francis E. Martin, of Massachusetts, read an instructive paper on "The Propagation, Preservation, and Use of Vaccine Virus."

The closing address was delivered by Dr. Eugene Foster, of Augusta, Ga., entitled, "The Statistical Evidence of the Value of Vaccination to the Human Race."

The celebration was under the auspices of the American Medical Association and of the American Public Health Association.

SECTION ON SURGERY AND ANATOMY.

The Chairman, Dr. C. A. Wheaton, of St. Paul, in making the annual address, reviewed at some length the history of this section of the Association, and mentioned the names of many men who have been prominent in its work in years gone by. He deplored the fact that many of these men too often absent themselves from these meetings, as it deprived the section of their counsel and assistance, which is so absolutely essential to its success.

Dr. Cark Beck read a paper on "Subphrenic Abscess in its Relation to Pyothorax." He stated that of five cases of subphrenic abscess, which he had observed, only twice was he able to make a correct diagnosis before operation. The fact that subphrenic abscess is very often confounded with pyothorax is well known, and such errors impress one gravely with the necessity of widening his limited diagnostic knowledge upon the subject. In referring to the treatment, the author stated: "The history is the most important guide in differentiation. In subphrenic abscess there is generally a history of a previous abdominal disturbance. There is no history of cough and expectoration as in pyothorax. The heart is little, if at all, displaced, and there is no ectasy of the thorax or of the intercostal spaces. In the lungs vesicular breathing is found below the clavicle; the pectoral fremitus is also clearly perceptible. There is a well-marked limit to the region of vesicular breathing below which the expiration murmur is replaced by amphoric sounds. Deep inspiration pushes the boundary line of the vesicular breathing much further down into areas in which formerly no respiratory murmur could be perceived. This would indicate a well-marked separation between the lungs and the abscess cavity, the boundary line of the lungs protruding towards the abscess cavity during deep inspiration. It is sometimes impossible to distinguish an encysted pyothorax from a subphrenic abscess. The pathognomonic signs of such effusions urged by Leyden were absence of cough and expectoration, slight displacement of the heart and rapid change of note if the patient is rapidly turned. According to the author's observations, however, pleuritic effusion, particularly pyothorax, sometimes occurs without these symptoms."

Dr. Alexander Hugh Ferguson, of Chicago, read a paper upon "Thoracoplasty in America (Schede's) and Visceral Pleurectomy, with Report of Cases." Thoracoplasty, as first done by Schede, is an heroic measure for the otherwise hopeless cases of chronic empyema, and consists of the removal of the chest wall. Dr Ferguson described his method of operating, and stated that some cases are not cured in spite of any operation performed for their relief. In his opinion, the fact that any amyloid degeneration and tuberculosis does not contraindicate this operation, as stated by Schede, is only true within certain limits. The author first performed Schede's operation in July, 1895, in which case a good result was obtained. Healing by first intention was secured, and the patient was able to be out in a very short time. In spite of careful treatment for five months after the operation, owing to the fact that a long central sinus had not closed, the operation of visceral pleurectomy was performed, which resulted in the patient's complete restoration to health. This operation has been performed by several men in America since it was first instituted in this country in October, 1893, by Dr. Geo. R. Fowler, of Brooklyn.

Dr. Howard Kelly, of Baltimore, made some remarks concerning his method of treating Extrauterine Pregnancy.

He spoke of several causes for error in the diagnosis and treatment of this affection, and mentioned that he had mistaken for extrauterine pregnancies dermoid cysts, pelvic abscesses, and small ovarian cysts, all of which he evacuated through the vagina. In his opinion, it is rarely necessary to open the abdomen for this purpose.

"Cholelithiasis and Cholelithotomy," by Dr. Charles H. Dunn, of Minneapolis. This paper was based upon a study of forty cases of gall-stone disease and a review of the chief literature upon the subject. In the author's opinion the indications for the operation of cholelithotomy were imperative.

Dr. Donald McLean agreed with Dr. Dunn, and stated that a successful operation on the gall-bladder gave gratifying results.

Dr. A. H. Ferguson, of Chicago, considered that the most important question was when to operate. When the gall-bladder was found enlarged so that apparently there was a tumor present, an operation should be performed.

Dr. W. J. Mayo, of Rochester, Minn., read a paper entitled "Some Mechanical Causes of Interference with the Action of the Stomach and their Surgical Relief." He divided the subject into two classes: *First*, those which act from within the cavity of the stomach or its immediate connections, such as a tumor, cicatrix, or a foreign body which may obstruct its inlet or its outlet, or prevent its normal muscular action; and, *second*, those which act from without the stomach and interfere either by pressure or adhesion, obstructing its inlet or outlet, or fixing some portion of its wall, thus preventing its functions. The main diagnostic resources are the physical examination, the distention with air, and the test-meal. The author advises Fenger's oblique lateral incision through the abdominal walls, in order to perform gastrotomy, for the purpose of retrograde dilatation; Witzel's method for temporary purposes, and Frank's spout method for permanent feeding.

Dr. W. P. Nicolson, of Atlanta, read a paper on "Ligation of the External Carotid Artery." This operation was done in conjunction with exsection of the jaws and of the inoperable diseases of the same.

Dr. M. M. Johnson, of Hartford, Conn., read a paper on the "Technique of Removing the Appendix Vermiformis." One hundred cases were reported. The author described the method of preparing the patient for the operation, which he is in the habit of employing in these operations. He strongly advised as little manipulation as possible of the intestines. In chronic recurrent cases he makes an incision 1½ inches long directly over the appendix, and when the peritoneum is reached a catgut suture is passed through it at the upper angle of the incision so as to prevent its retraction. In finding the appendix he passes the thumb and index finger through the incision, seizes the colon, which he draws through the incision until the appendix is brought into view. When the cecum is surrounded with a pus sac, a perforated and sloughing appendix is usually found and should be removed. It should be a fixed rule never to close the incision without finding and removing the appendix. The treatment of the stump resolves itself into the treatment of an intestinal perforation and the surgical rule for the treatment of this condition should not be violated in the treatment of the stump.

With regard to the time for operation, this should be decided from the standpoint of the best interests and safety of the patient. In the author's opinion, operative measures should be an early consideration rather than a last resort. Of 100 operations performed by Dr. Johnson, 98 resulted in recovery and 2 in death, of which 64 were males and 36 females.

Dr. Parker, of New Orleans, could not agree with the author as to the advisability of removing the appendix at all times. In those cases where the appendix is surrounded by an abscess, all that is necessary is to open the abscess and drain the region.

Dr. A. H. Ferguson said the most favorable time to operate was between the attacks, and he believes it dangerous to operate during the acme of the inflammation. The best way of draining these cases is with gauze and a glass tube.

Dr. Hunter P. Cooper, of Atlanta, read a paper entitled "Gastrostomy for Stricture of Esophagus—Sbanijeu-Frank Method," and exhibited the patient. He related the history of a young man who, one year ago, took one swallow of pure nitric acid by mistake, causing corrosion of the entire mouth, lips, and esophagus. The mouth and lips healed about three weeks after the slough separated. The author first saw the patient in January, 1896, and several months previous to this an increased difficulty in swallowing was experienced, but at this time this symptom was considerably worse, and the feeling existed that small particles of food were stopped before reaching the stomach, evidently low down in the esophagus. Liquids could be swallowed slowly and with difficulty, half an hour being occupied in drinking a glass of milk. Examination of the esophagus revealed three strictures, seven, seven and a half, and eight inches from the edge of the incisor teeth. No more were discovered until the instrument was passed thirteen and a half inches, when an obstruction was met with, which would not allow any instrument to pass. The largest instrument that would pass the stricture seven inches from the teeth was of the size of a 22-French urethral bougie, and nothing would pass lower than thirteen and a half inches. Gastrostomy was decided upon and the Sbanijeu-Frank method was selected, which the author briefly described. The advantage of this method is that it brings the stomach through the abdominal wall in an oblique direction, and a part of the stomach is included between the edge of the costal cartilage and the skin, forming a valve. This device prevents leakage of the contents of the stomach during digestion and is a great advantage over the old method of making a direct communication between the stomach and the abdominal incision. The subsequent history of the case was uneventful, the patient gaining in flesh and strength and being perfectly nourished by means of feeding through the artificial opening.

Dr. F. W. Epley, of New Richmond, Wis., read a paper on "Skin Surgery," in which he mentioned a case, having transferred 52 square inches of skin in six large pieces from five different persons, and 300 smaller grafts from four other persons, to repair an injury to a man's foot, all of which were successful. After a brief description of the method employed, he laid especial stress upon the necessity of using antiseptics freely, cocainizing fearlessly, and making the donor's wound boat-shaped.

Dr. Z. J. Lusk, of Warsaw, N. Y., mentioned a number of cases of skin-grafting, and stated that he had employed two methods in the work. The first method was that of raising epidermis by cantharides, and the second was the employment, after proper softening and sterilization, of exfoliated epithelium. In all cases in which these methods had been employed excellent results were obtained.

SECTION ON DISEASES OF CHILDREN.

The Chairman, Dr. A. C. Cotton, of Chicago, read an address entitled, "Has the Milk Laboratory Come to Stay?" The speaker had been guided somewhat in the selection of his subject by the recent interest taken in the elaboration of infant food. There are three defined classes of infant feeders: (1) Those who regard the infant as belonging to the carnivora; (2) those who would place the nursling of the human species among the granivora, and (3) those who early place the young in the omnivorous class. Says one theorist, "Raw milk is poison"; another, "Bottled milk is indigestible"; another, "Starch is indigestible," and still another, "Cereals are unnatural." The speaker believes that the milk laboratory has come to stay. The advantages lie in the direction of more exact manipulation, greater precision and accuracy in the keeping of records.

Dr. J. A. Larrabee, of Louisville, followed with a paper on "Pediatric Therapeutics as Proven by Experience." He said widely different effects may be obtained from the same drug in different doses. In children the best are usually to be obtained from small doses, frequently repeated. In the discovery of new remedies we have been more successful reasoning by synthesis than by analysis. The author condemned the use of polypharmic compounds, emphasizing the importance of the use of simple prescriptions.

He believes the use of antiseptics good practice. Thus, he has obtained good results with listerine and borolyptol in infants with lactic acid fermentation in the stomach, as well as in children with putrefactive changes in intestinal ingesta, after removal of ingesta by enema and purgation. Guaiacol has proven to be the ideal intestinal antiseptic.

Dr. Elmer Lee, of Chicago, read a paper in which he followed quite closely the statistics and arguments of Dr. Winters, of New York, in opposing the use of antitoxin. Horse-serum poisoned by germ culture has no natural place in the human blood. The red blood corpuscles are dissolved whenever horse-serum comes in contact with them.

Dr. J. W. Stickler, of Orange, N. J., concluded from his experience that when the heart's action is fairly good, when the system has not been seriously impaired, when the urine shows little or no albumin, antitoxin, judiciously used, is likely to benefit the patient, but when there is great depression of the vital forces it is contra-indicated.

Dr. Henry E. Tuley, of Louisville, read a paper on "Sepsis of the Newborn." In this he included the report of a case in which the mother, who was a healthy woman, had been in a well-appointed hospital and under the supervision of competent physicians and trained nurses for several weeks before confinement. The latter was normal and followed by an uneventful recovery. The child was healthy until an infection occurred at the umbilicus in spite of the most carefully conducted dressings which were applied. The author made the suggestion that this case should not lead any obstetrician to relax his careful use of aseptic dressing of the cord.

The subject of "Chorea" was introduced by a paper by Dr. Henry Hatch, of Quincy, Ill. He believed the nervous affection was directly due, in the majority of cases, to the modern faulty methods of rearing and educating girls especially. He suggested a modified rest treatment, combined with tonics, such as rapidly increasing doses of strychnia.

SECTION ON NEUROLOGY AND MEDICAL JURISPRUDENCE.

Dr. T. D. Crothers, of Hartford, Conn., the Chairman of the Section, presented the usual address, in which he commented upon the growing importance of neurology, and the legal relations and possibilities constantly growing out of present conditions, and spoke of the numerous publications on this subject during the past year. The present treatment of crime, insanity, and drug manias by legal methods has not kept pace with the march of science, so that it is no wonder that expert testimony has fallen into disrepute.

Dr. J. T. Searcy, of Tuscaloosa, Ala., read a paper entitled "Intoxication and Insanity." He limited the meaning of the word to the injurious effects on the cerebrum of toxic agents present in the circulation, the symptoms of intoxication being those that belong to the brain, this organ being exceedingly sensitive to the action of certain agents. Cerebral intoxication arises in the person according to the character of the agent and the particular brain; that is, according to the toxin and according to the abnormality of the particular brain.

Dr. G. W. Drake, of Chattanooga, read an article entitled "Man's brain and Mind: The Former Sometimes Insane, the Latter Never." The author held that a sane brain is one in which all its cells and cell derivatives are in a normal state chemically, physically, and vitally. The mind is never insane. Its brain machine may break down and need adjusting, but the mind cannot be injured by traumatism, auto-intoxication, or bacterial toxins.

A paper entitled "A Note on the Patho-Mental Effects of Degenerative Habit" was read by Dr. H. S. Drayton, of New York City, in which he referred to Maudsley's statement that a man is "by no means hopelessly chained down by the weight of his inheritance, for there is something else that makes fate, and that is education." Habits, however acquired, produce in time conditions of mind and body which of themselves reflect a healthful or injurious nature.

Dr. F. Peterson, of New York, read an article on the "Medical and Surgical Treatment of Epilepsy," in which he summarized his remarks as follows:

1. In about 1 per cent. of all cases of epilepsy an injury to the head will be found to be the original cause.

2. In a much larger percentage, an old meningeal hemorrhage, congenital or acquired in infancy, giving rise, in addition to the epilepsy, to various degrees of paralysis, idiocy, or other cerebral symptoms, and presenting on examination atrophic, sclerotic, or cystic sequelæ to the primary lesion, will be found to be the cause.

3. Cases due to the meningeal hemorrhage should not be operated upon.

4. In the very small number of cases having injury to the head as a cause, the epileptic habit is so strong and the changes in the brain usually so old and so deep-seated that operation does not cure and only seldom permanently diminishes the frequency of attacks.

5. Of miscellaneous traumatic cases where surgical procedure seems justifiable and is undertaken, a cure may be reasonably expected in perhaps four out of every hundred cases operated upon.

6. The removal of a cicatrix from the cortex supposed to be the epilepto-genetic nidus will naturally be followed by the formation of a new cicatrix in the surgical wound.

7. If a hundred cases of epilepsy could be selected in which the trauma dates but a few months back, trephining and ablation of the morbid tissues would doubtless prove curative in a very large percentage of cases.

Dr. T. O. Powell, of Milledgeville, Ga., read a paper on "The Increase of Insanity and Tuberculosis in the South." He said the negroes of Georgia and the South in general, up to 1860, enjoyed remarkable mental and physical health, and were almost entirely exempt from certain diseases, owing to the care of their owners and the hygienic precautions taken by the latter. In 1860 there were only forty-four insane negroes in the State of Georgia, the majority of whom had white blood in them. In 1870 there were 129 insane negroes in the State, in 1880 411 insane negroes, and in 1890 910 insane negroes in a population of 858,815, or one colored insane to every 943 of population.

Dr. John Punton, of Kansas City, Mo., read a paper on "The Etiology and Prophylaxis of Functional Nervous Diseases," in which he referred to the advance in our knowledge of the anatomy and physiology of the nervous system. Every practitioner recognizes that nervous diseases are increasing at a marvelous rate. The duty of the physician is to use all possible means to secure the needed rest for all morbid conditions of the nervous system, in which nervous irritability and nervous instability are the chief clinical criteria.

Dr. A. C. Corr, of Carlinville, Ill., read a paper entitled "The

Medical Aspects of Crime." The brain and the nervous system are the physiological organs of the mind and intellectuality, and every grouping of ideational activity has its origin in the functional activity of brain and nerve cells. In order to lessen crime we must cultivate and develop the moral sense and repress the emotions. Children should be taught as early as psssible that there is a principle of right and justice in the abstract, irrespective of any mere religious sentiment. The moral defect, with its criminal tendencies, is always congenital, often hereditary, and always modified in a greater or less degree by environment and synergistic influences.

"The Physician and the Criminal." A paper was read on this subject by Dr. J. B. Ransom, of Clinton, N. Y. The author commented upon the dearth of records as to the study of criminal man. The alarming ascendancy of crime should excite society to vigorous effort. The modern trial, sentence, and punishment have failed. The procedures of the courts and administration of law are based upon the theory that crime must first be detected and then punished, losing sight of the fact that men are not alike, and that punitive measures should be adapted to the individual characteristics of the criminal. He estimates that 25 per cent. of all criminals would show disease of the heart or of the great blood-vessels. Meningitis is also a prolific cause of crime.

Dr. D. R. Brower urged the profession to enlist in a crusade for the more rational treatment of crime. He favorably commented upon the custom of indeterminate sentences, whereby the criminal was induced to improve by the prospect of release added to the fear of continued imprisonment.

### SECTION ON OBSTETRICS AND DISEASES OF WOMEN.

The meeting was called to order by the Chairman, Dr. Joseph Taber Johnson, of Washington, D. C., who read an address upon the "Recent Progress in Obstetrics and the Diseases of Women." He first considered puerperal infection, its causation and treatment, saying that this form of infection had been the cause of much discussion during the year. While something has been added to scientific knowledge of the history and behavior of certain pathogenic germs, the general course of treatment of these cases has been simplified and shortened. In the treatment the best instrument is the index finger, with which portions of after-birth or membranes can be removed, using, if necessary, bimanual pressure under ether. As regards hysterectomy for puerperal infection, it seems to be gaining ground, notwithstanding the opposition of Lusk, Price, and a number of other prominent men in this country and abroad, though the cases where it is indicated seem to be very few. The next subject considered was placenta previa. Nothing new seems to have been added during the year, except the suggestion that hysterectomy be done for the control of the hemorrhage. The diagnosis is generally not made until the occurrence of the latter.

Dr. Henry P. Newman, of Chicago, read a paper entitled "A Note on Stenosis of the Cervix as a Factor in Uterine Disease." He said that twenty years ago this subject was one of the topics of great interest among gynecologists. The literature abounded with learned and exhaustive treatises upon the pathology and treatment of the affection. Notwithstanding the amount of literature formerly written upon this subject, the hundreds of operations recorded by older gynecologists, and the nicely adapted instruments that remain as evidences of the reality of stenosis of the uterine cervix, there are some modern authorities who deny the existence of this affection as an anatomical fact. Stenosis was defined as a narrowing of the caliber of the canal, and may be situated at the external os, the internal os, or may include the whole extent of the canal. This condition causes sterility and dysmenorrhea by offering a mechanical obstruction to the entrance of the fecundating element, and by preventing the free discharge of the menstrual fluid.

Dr. I. S. Stone, of Washington, D. C., read a paper entitled, "How to Remove Pus Tubes without Rupture." He illustrated the method by exhibiting a diagram showing the application of clamps to uterine and ovarian vessels before enucleation from the anterior surface of the abscess wall. He summarized the application of this method as follows: It gives more space for careful enucleation. The larger blood supply is tied off before enucleation begins, and no severe hemorrhage can occur during or after the operation. Smaller silk or catgut can be employed, as no *en*

*masse* ligatures are used. If rupture is avoided, there is no need of flushing or drainage. It facilitates enucleation by affording another point of cleavage, namely, from anterior surface of the pus sac. It facilitates exsection of the uterine cornua. Dr. Stone exhibited a huge pyosalpinx, nearly nine inches in circumference and fourteen inches in length, which he had recently removed after the manner described in his paper.

Dr. W. G. MacDonald, of Albany, read a paper upon "The Present Status of Ectopic Pregnancy—a Surgical Disease." He said the surgery of ectopic pregnancy is mature rather than old. The pathology and principles of treatment are already established. Yet women are dying every day from ruptured ectopic pregnancy with no effort being made to rescue them because the condition is not recognized during life. Diagnosis only follows careful study of the clinical history and painstaking physical examination. There are two general propositions which should be generally appreciated in the diagnosis of ectopic pregnancy. *First*, any woman who, during her child-bearing period, presents herself with symptoms of disease of the organs of generation, of recent origin, either new or entirely different from those heretofore experienced, if associated with any of the early symptoms of pregnancy, demands at once a careful examination. *Second*, abdominal pain, either continuous or intermittent, is always an important symptom and requires the fullest investigation.

The treatment of ectopic pregnancy is surgical. Operation is to be undertaken as soon as a diagnosis is established. Many cases are emergency surgery, and require immediate operation. Complete tubal abortion, if diagnosticated, will seldom call for surgical intervention.

Dr. Joseph Price, of Philadelphia, followed with a paper entitled "Symptoms, Diagnosis, and Time for Operation in Ruptured Tubal Pregnancy." He said that the occurrence of tubal pregnancy is regarded in widely different lights by the theorist and the surgeon who has learned to deal with it practically, and who has accordingly come to understand the manifold directions in which speedy disaster may swoop down upon unfortunate women subjected to this calamity. As to the causes of aberrant gestation, we are to consider them both as anatomical and moral. Causation can rarely be determined with certainty. There are many agencies which operate to produce the trouble. The character of the attack, the whereabouts of the patient, her employment, are always interesting considerations. Dr. Price is convinced, fortified by his own experience, counting now 128 cases with five deaths, that the operative treatment is the only one to be considered. He is fully satisfied that these pregnancies are rarely in the broad ligament. He regards hemorrhage as the chief danger of the operation.

Dr. Milo B. Ward, of Topeka, Kansas, read a paper entitled "Gauze as Drainage in Abdominal and Pelvic Surgery." He said it was idle to say that drainage is needed only when the work has been carelessly done. However perfect the technique may be, it is still a fact that there are cases whose recovery depends entirely on the drainage employed after the surgical toilet is made. The author urged the importance of the thorough use of gauze in a large variety of complications, believing it to be the material which met the most indications.

Dr. Howard A. Kelly no longer drains in cases of tubercular peritonitis, and the cases practically all recover. The cases that he drains usually have fistulous tracts. When he does drain he uses gauze.

Dr. Charles P. Noble, of Philadelphia, said the more he operates the less he drains. At the present time he drains but little. When he uses gauze for drainage purposes he also uses a glass tube in addition to remove the fluid from the gauze, believing that gauze of itself does not drain.

Dr. H. O. Pantzer, of Indiana, said that the peritoneum was amply able to take charge of a limited amount of pus, when the affection of tissues was not too great. He thought gauze packing was unnecessary in a great many cases. He suggests the use of peroxide of hydrogen to loosen the gauze from its attachments where the gauze is used for drainage purposes.

Dr. A. P. Clarke, of Cambridge, Mass., read a paper entitled "Degenerative Changes that Occur in Uterine Fibro-myomatous Growths." The author begins by saying that the presence of uterine fibroid growths has for the past few years given rise to much discussion among gynecologists. The consideration of the surgical treatment which they have demanded has received much attention, and measures of procedure, in some instances apparently opposite, have sometimes been adopted. The success attending the removal of fibroid growths either by partial or by total hysterectomy has brought the subject of such neoplasms more prominently before the profession.

Aneurismal and also fatty degenerative changes were spoken of and discussed at some length. Another form which has a practical significance to the abdominal surgeon is that resulting from the occurrence of enormously enlarged vessels or capillaries. These vascular elements may develop at the expense of the connective and the muscular tissues. The several kinds of secondary transformations occurring in fibro-myomata are productive of further expulsive processes that often require only timely surgical interference as measures supplementary to the effecting of their complete removal.

"Drainage versus Radical Operation in Cases of Large Pelvic Abscess" was the title of a paper by Dr. Chas. P. Noble, of Philadelphia. He called attention to the value of drainage in the treatment of large pelvic abscesses, and contrasted the results which can be secured by this method of treatment with those which have been obtained by the more radical operation of abdominal section, together with the removal of pus sacs, whether of ovarian, tubal, or other origin. An ordinary case of pyosalpinx, or abscess of the ovary, chronic in character, was ruled out of consideration.

Until June 11, 1895, the author had never employed drainage without abdominal section in the treatment of this class of cases, but had always operated on them by abdominal section and the removal of the diseased parts. Since this period the speaker had operated upon eight cases without a death.

Dr. H. D. Thomason, of Albion, Mich., followed with a paper on "Some Suggestions in the Prophylaxis and Management of Puerperal Eclampsia." He first dwelt upon the pathology and etiology of puerperal eclampsia, saying that the etiology was still an unsettled question. As soon as he is satisfied of the viability of the child, he believes he is not only justified, but it is his duty to bring about premature labor, *first*, upon the ground of safety, welfare, and comfort of the mother; *second,* for the welfare and safety of the child.

"Intestinal Obstruction after Laparotomy" was the title of a paper by Dr. Henry O. Marcy, of Boston, in which, before entering into a general discussion of this subject, he gave a brief report of all the cases of intestinal obstruction following laparotomy which had come under his observation. He reported seven cases.

Dr. R. R. Kime, of Atlanta, read a paper entitled "Puerperal Infection; Its Pathology, Prevention, and Treatment." The author stated that prevention of puerperal infection is accomplished by strict asepsis in obstetric practice, as well as thoroughly emptying of uterus and securing proper contractions with repair of lacerations.

"Gonorrhea in the Puerperium," by Dr. A. H. Burr, of Chicago. In an obstetrical experience of 500 cases the author has had three fatalities, each of which was directly traceable to gonorrhea. In many of the remaining cases of puerperal sepsis, more or less severe, he had found by clinical history or by microscopic verification, the presence of a gonorrheal infection.

Dr. Reuben Peterson, of Grand Rapids, Mich., read a paper on "Hysterectomy as an Accompaniment to Bilateral Removal of the Appendages." The author made a plea against the adoption of the universal rule in regard to these cases as if it were for all time settled. He claims that the surgeon has no right to remove the uterus after removal of the appendages unless he is convinced that the organ is diseased beyond the hope of cure by less radical methods.

### SECTION ON THE PRACTICE OF MEDICINE.

The Chairman, Dr. Wm. E. Quine, of Chicago, read an address upon the "Advances in Therapeutics during the Year," in which he spoke of the therapeutic effects and *modus operandi* of bone marrow, and he was of opinion that the agent was of undoubted value in relation to the treatment of ordinary simple anemia and chlorosis, and in cases of anemia of the more intractable kind. Fourteen cases of Addison's disease treated with the adrenals of lower animals, mostly those of sheep, are now on record. The favorite preparation appears to be a glycerite of the minced organs, given with a degree of freedom amounting to from fifteen grains to three drams of those organs daily. In about one-half of the recorded cases, improvement has been noted, and in a few of them the improvement has been very striking, but we are lacking in information as to the stability of it.

Dr. J. W. Grosvenor, of Buffalo, read a paper on "The Effect of Alcohol on the Organs of Special Sense," in which he took the position that upon these organs alcohol is a paralyzer and depressant even when given in small doses. The popular doctrine that alcohol is a stimulant is erroneous, and the resort to it for its supposed stimulant action in cases of cardiac depression, or heart failure, or during anesthesia by chloroform or ether, is inadmissible and based upon wrong views.

This radical opinion was vigorously opposed by Drs. Hare, of Philadelphia; Haine, of Chicago; Ouchterlony, of Louisville, and Hill, of Milwaukee.

"Early Diagnosis of Cancer of the Stomach by Means of Chemical Analysis of the Gastric Contents" was the title of a paper by Dr. W. C. Weber, of Cleveland, Ohio. The absence of hydrochloric acid in the stomach contents is not evidence of cancer. Neither is the presence or absence of lactic acid reliable. He advised microscopic examination of the blood, and careful palpation, percussion, etc., in order to make out a diagnosis.

Dr. Everett Flood, of Massachusetts, presented a paper on "Intestinal Antisepsis, Diet, and Castration in Relation to the Treatment of Epilepsy." He described some illustrative cases in which the operation had been followed by some benefit.

Dr. J. M. Marvin, of Louisville, read a paper on "The Ductless Glands," calling attention to the economic importance of the "internal secretion" of these glands. He said that the secretions of the suprarenals, thyroid, pituitary gland, when emptied into the blood influences nutrition, and that associated with perverted secretory functions of these glands we have Addison's disease, exophthalmic goitre, myxedema and cretinism, acromegalia, etc. So, also, interrupted functions of the bronchial glands may have something to do with the clubbed fingers and other nutritional changes in the skin and bony tissues in the later stages of pulmonary tuberculosis.

The Committee on Nominations reported the following officers for the ensuing year:

*President*—Dr. Nicholas Senn, Chicago.

*First Vice-President*—Dr. George M. Sternberg, Surgeon-General U. S. Army, Washington, D. C.

*Second Vice-President*—Dr. Edmond Souchon, New Orleans.

*Third Vice-President*—Dr. J. B. Thomas, Pennsylvania.

*Fourth Vice-President*—Dr. Willis F. Westmoreland, Atlanta.

*Treasurer*—Dr. H. P. Newman, Chicago.

*Assistant Secretary*—Dr. T. B. Schneideman, Philadelphia.

*Librarian*—Dr. George W. Webster, Chicago.

*Chairman of Committee of Arrangements*—Dr. H. A. Hare, Philadelphia.

*Address in Surgery*—Dr. W. W. Keen, Philadelphia.

*Address in Medicine*—Dr. Austin Flint, New York.

*Address in State Medicine*—Dr. Jerome Cochran, Alabama.

The President-elect, Dr. Senn, was then escorted to the stage by a committee of two, presented by Dr. Cole, and made a timely and eloquent speech of acceptance.

After the introduction and adoption of resolutions of thanks, the Association adjourned to meet in Philadelphia the first Tuesday in June, 1897.

---

A SOCIETY in Paris, consisting of scientists of note, having about one hundred members, several of whom are women, has the ghastly purpose of placing the brains of its members at the disposal of surviving comrades, for examination and dissection. The society is known as the Mutual Autopsy Society (*La Société d' Autopsie mutuelle*), and was organized in 1876. Fourteen brains, neatly catalogued, are now contained in a glass case at the end of the meeting-room, and the fifteenth, which in life was the property of M. Abel Havelacque, director of the Anthropological Society, now rests immersed in alcohol on the table of the dissecting-room, where the man's former associates will meet to weigh, probe, cut, and discuss it.—*Medico-Surgical Bulletin.*

